# Cooperation and Conflict in the Plant Immune System

**DOI:** 10.1371/journal.ppat.1005452

**Published:** 2016-03-17

**Authors:** Eunyoung Chae, Diep T. N. Tran, Detlef Weigel

**Affiliations:** Department of Molecular Biology, Max Planck Institute for Developmental Biology, Tübingen, Germany; THE SAINSBURY LABORATORY, UNITED KINGDOM

Plants have a sophisticated innate immune system with which they defend themselves against a myriad of pathogens. During the past two decades, work in a range of species has advanced our knowledge of the molecular and biochemical details of plant immunity. Many of these studies have focused on the action of nucleotide-binding domain/leucine-rich repeat (NB-LRR or NLR) immune receptors. NLR genes constitute the most diverse gene family in plants, reflecting their role in perceiving a very diverse set of molecules that are released by pathogens. There has also been progress in unraveling the forces that drive diversification of NLR and non-NLR immune receptors in wild species. A major recent insight from mechanistic and evolutionary studies is that there is both cooperation and conflict in the plant immune system. Here, we propose that these two antagonistic forces are inherently entangled, and that they are potentially fundamental to our understanding of growth-defense trade-offs.

## Cooperation in the Immune System

Genetic studies of plant immunity have been tremendously influenced by Flor’s gene-for-gene hypothesis, which posits that a single host resistance gene is matched by a single effector gene from a specific pathogen strain. It was thus unexpected when two NLR genes that are located immediately adjacent to each other in the *Arabidopsis thaliana* genome were found to cooperatively confer resistance to the *Hyaloperonospora arabidopsidis* ex *parasitica* isolate Cala2 [1]. Since then, several more such pairs have been discovered, with one member often encoding other protein domains in addition to the canonical “NLR” moieties [2]. Two good examples are the RPS4/RRS1 protein pair, which endows *A*. *thaliana* with resistance to both bacterial and fungal pathogens, and the RGA4/RGA5 pair in rice, which confers resistance to specific races of the fungus *Magnaporthe oryzae*. The dual genetic requirement translates into direct physical interaction, with RPS4 and RRS1 (as well as RGA4 and RGA5) forming heterodimers [2–5]. In both cases, one protein, RRS1 or RGA5, contains an additional specific recognition domain at the C-terminus [2,6]. Binding of a pathogen effector protein to this domain alters the interaction with its respective partner, RPS4 or RGA4, and thereby leads to host immune signaling. The addition of an effector-binding domain to one of the NLR proteins may have facilitated expansion of recognition specificity, since the evolution of the recognition domain is not necessarily coupled to constraints acting on the NLR moiety.

An attractive hypothesis is that the involvement of two physically interacting NLR proteins not only increases the specificity of the response compared to effector recognition by a single protein but also provides safeguards against inappropriate activation. In support of such a scenario, ectopic expression of RPS4 or RGA4, but not of the effector-detecting RRS1 or RGA5, causes autoimmunity [2,3,7]. The tight physical linkage in the genome would support functional coevolution, as seen in other clustered immune-gene systems (e.g., [8]). This may also explain why functionally dependent but unlinked NLR pairs are less common [9].

## Conflict in the Immune System

Another common type of protein–protein interaction in the plant immune system is the one between immune receptors (often NLR proteins) and host clients that are direct targets of pathogen effectors. From the host perspective, pathogen-targeting of the NLR clients may be undesirable when it leads to enhanced pathogen virulence; in this case, clients are considered NLR-guardees. In other cases, however, targeting may be desirable, because the NLR-clients are merely decoy versions of true targets [10]. The interaction between the NLR-guard and its guardee/decoy mediates indirect recognition of pathogen effectors [9]. Similar to the NLR–NLR interaction discussed above, the guardee/decoy partner modulates signaling of the NLR protein; if the guardee/decoy is compromised, either by effector-dependent modification or mutation, immune responses are activated. This can also occur when the NLR (or a non-NLR immune receptor) and its host client have not coevolved and are mismatched [11,12]. Such spontaneous, pathogen-independent autoimmune reactions can be observed in many genetic intra- and interspecific crosses, a phenomenon that breeders have known as hybrid necrosis for decades [13].

*A*. *thaliana*, an excellent species for the study of naturally occurring genetic variation, has proven to be valuable for the investigation of hybrid necrosis. The first report of a hybrid necrosis locus in *A*. *thaliana*, *DANGEROUS MIX1* (*DM1*), identified an NLR protein that triggers autoimmunity when combined with a specific allele at the unlinked *DM2* locus [14]. A recent systematic study using thousands of intraspecific *A*. *thaliana* crosses has not only corroborated NLR loci as major contributors to hybrid necrosis but also confirmed that the genetic architecture of hybrid necrosis is often simple, with typically one or two causal loci. Notably, several cases involve NLR–NLR interactions, including the DM1/DM2 case [15]. NLR genes show extreme sequence and copy number variation both within and between species [16], reflecting the multitude of pathogen molecules that are directly or indirectly detected by NLR proteins. It thus hardly seems surprising that such extreme diversity can lead to collateral damage in the form of hybrid necrosis—highlighting a conflict between diversification of the immune system and avoidance of unwarranted autoimmunity.

Another outcome of this conflict appears to manifest itself as the suppression of host resistance, which has been observed during attempts to pyramid valuable resistance genes in crops. At least in one such case, in wheat, resistance suppression results from mismatched interactions between NLR proteins that are encoded by different alleles of the same locus [17,18]. Given that other NLR protein pairs positively cooperate for proper function [2,3], an obvious hypothesis is that resistance suppression is caused by inappropriate physical interactions between the different NLRs. Such opposite outcomes of interference among plant immune system components—either leading to hybrid failure due to hyperimmunity or to weakened immune response—had previously been predicted from modelling the effects of pathogen diversity on the evolution of interactions within the plant immune system [19].

## Towards Dissecting Trade-Offs between Growth and Immunity

Individual NLR genes can incur substantial fitness costs in the absence of the pathogen they recognize, not only in the greenhouse but also in the field (e.g., [20]). Ideally, immune receptors would, of course, be perfectly “off” in the absence of a trigger. The fact that there are apparent NLR effects, even without pathogen effectors, points to a biochemical trade-off between the robustness of the on/off switch and sensitive, highly specific activation of immune receptors. Still, knocking out common components of NLR downstream signaling does not lead to enormous increases in growth. One can think of several explanations for why this is the case; for example, it could be that NLR genes with substantial fitness costs are the exception, with aberrant NLR activity being often suppressed by other NLR genes, or that there is an inherent limit to the resources that will normally be diverted to defense upon NLR activation. No matter what the right answer is, we certainly need a better understanding of the trade-offs both within the immune system and between immunity and growth. One approach is to investigate biochemical details of immune receptor activation, and of the connection between immune receptors and hormone signaling [21]. Similarly, phenotypic differences among different autoactive NLR mutants (e.g., [22]) and between different hybrid necrosis cases [15] may be illuminating in this regard.

**Fig 1:**
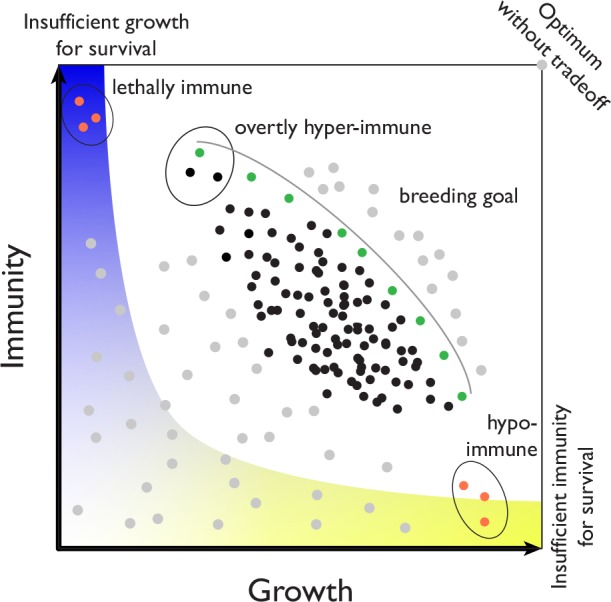
Hypothetical phenotypic space determined by trade-offs between growth and immunity. Common, reasonably fit genotypes are in black; genotypes that could potentially be created by crosses or mutations are in grey and orange. Green are genotypes closest to the Pareto front (grey line), which defines optimality among existing genotypes. The blue/yellow area represents forbidden phenotypic space, i.e., genotypes that die, always succumb to pathogens, or are infertile. The Pareto front can shift in response to a certain pathogen pressure or to abiotic challenge.

A complementary approach begins with a population perspective. We hypothesize that the strong necrosis phenotypes seen in some hybrids constitute the proverbial tip of the iceberg of hybrid immune reactions. A corollary of this proposal is the ubiquity of more subtle epistatic interactions that affect both the state of the plant immune system and growth or fertility. Systematic analyses of intraspecific crosses in *A*. *thaliana* have already shown that subtle F_1_ hybrid necrosis cases are much more common than drastic, lethal interactions [15], and there are hybrid necrosis cases that are only revealed in the F_2_ generation [23,24]. We like to think about the trade-offs in the framework of phenotype space (Fig 1). In addition to the lethally immune and hypo-immune hybrid genotypes discussed in the previous section, there are natural genotypes that suffer from autoimmunity and reduced growth but, at the same time, benefit from pleiotropic broad-spectrum resistance against a wide range of pathogens [25].

Of most interest in this abstract phenotypic space is the “Pareto front” (grey line in Fig 1), which describes a classical optimization problem [26]. It is clear that the preferred genotypes are those that are closest to the Pareto front (colored green in Fig 1), but that the particular pathogen pressure will determine which of these preferred genotypes is superior in a specific situation. Given that, in reality, the phenotypic space (and therefore the position of the Pareto front itself) will vary with shifts in pathogen populations and abiotic conditions, it will be important to determine the Pareto front in numerous controlled laboratory settings as well as in natural habitats of *A*. *thaliana*. We also note that mild necrosis in either inbred *A*. *thaliana* accessions or progeny of crosses is not that rare [15,23,25], suggesting that mild necrosis risk alleles are maintained at an appreciable frequency. Important goals for the future are, therefore, to better understand how the inherent trade-off between growth or fertility and immunity is managed in natural populations, to find out whether one can extrapolate from findings in *A*. *thaliana* to other species, and to test whether lessons from wild plants such as *A*. *thaliana* can be useful in crop breeding.
